# Analysis of machining performance in turning with trihybrid nanofluids and minimum quantity lubrication

**DOI:** 10.1038/s41598-025-97039-7

**Published:** 2025-04-09

**Authors:** Javvadi Eswara Manikanta, Masuk Abdullah, Nitin Ambhore, Tarun Kumar Kotteda

**Affiliations:** 1 Department of Mechanical Engineering, Shri Vishnu Engineering College for Women, Bhimavaram, 534202 India; 2 Department of Vehicles Engineering, Faculty of Engineering, University of Debrecen, Ótemető strt. 2–4, Debrecen, 4028 Hungary; 3 Department of Mechanical Engineering, Vishwakarma Institute of Technology, Pune, 411037 India; 4 Department of Mechanical Engineering, Sagi Rama Krishnam Raju Engineering College, Bhimavaram, 534204 Andhra Pradesh India

**Keywords:** MQL, Trihybrid nanofluid, Machining, Surface roughness, Cutting temperature, Engineering, Materials science

## Abstract

Mono-type and dihybrid nanoparticle-enriched cutting fluids in machining processes are gaining popularity because of their outstanding benefits such as enhanced tool life and surface finish. In this work, a trihybrid nanocutting fluid was developed by mixing MWCNT Al_2_O_3_ graphene nanoparticles with different weight concentrations. The prepared tri-hybrid nanofluids were tested during the machining of SS304 steel. Coated tungsten carbide and PVD TiAlN carbide tools are used for the machining. Analysis of variance (ANOVA) and response surface methodology (RSM) were used to analysed the obtained data. ANOVA revealed that the interaction of cutting speed and feed had shown a good impact on surface roughness. The combinations of minimum quantity lubrication (MQL) and tri-hybrid nanofluid characteristics increase surface quality by 16% and the cutting temperature by 76%, respectively, which offers future applications in the machining industry. The correlations are verified using a conformance test and have acceptable variances of 3.11% and 1.13% with the ANN approach regression analysis.

## Introduction

In dry machining processes, due to elevated temperature levels in the machining zone, the cutting speed is limited^1^. Excessive heat further impairs the durability and cutting tool wear, resulting in early tool failure^2^. Consequently, the adoption of appropriate cutting fluids becomes very important to address these challenges in high-speed machining^3,4^. Traditional cutting fluids are helpful in lubrication and facilitating the removal of chips from the cutting zone^5^. While these fluids have served their purpose to a considerable extent, their overuse has raised environmental concerns and potential hazards to human health^6^. Additionally, the extensive use of traditional cutting fluids adds to production costs in the machining industry, necessitating a shift towards more efficient alternatives^7^. In response to these challenges, the concept of MQL has materialized as a promising technique^8^. Nanofluid-based MQL has recently gained significant attention because of its ability to enhance tool life, and surface quality and reduce environmental impact^9^. Haghnazari and Abedini^10^ investigated the machining performance of Al_2_O_3_ and CuO hybrid nanofluids in turning AISI 4340 steel with MQL system. The experimental finding revealed considerable enhancement in surface quality.

Das et al.^11^ evaluate the machining performance in turning of AISI H13 steel multi-walled carbon nanotubes mixed nanofluid with MQL. The finding revealed that surface finish is strongly affected by increased tool wear and production of segmented type serrated saw-toothed chips. Conventional cutting fluids, while offering noble lubrication properties, often fall short in terms of their thermal properties for industrial purposes^12^. Therefore, researchers have introduced nanofluids, incorporating nanometer-sized particles into traditional fluids to create a new generation of cutting fluids^13^.

Using hybrid nanofluid MQL, Usluer et al.^14^ estimated the optimum cutting parameters for turning S235JR structural steel. The findings revealed that the nanofluid MQL cutting environment reduces energy consumption and has better tool life than dry machining does. Research conducted by Kumar et al.^15^ and Virdi et al.^16^ confirmed that the incorporation of nanoparticles into cutting fluids boosts the conductivity, subsequently improving surface quality, and tool life with minimum cutting force and temperature. Furthermore, the results revealed that the use of graphite nanoparticles has shown lower friction^17^. Sharma et al.^18^ investigated hybrid nanofluids and results showed that wear decreased with an increase in nanoparticles in cutting fluid. Safei et al.^19^ discovered tri-hybrid nanofluids that effectively reduced cutting temperature and enhanced surface quality. Research by Ramadhan^20^, developed tri-hybrid nanofluids and found a reduction in tooling costs related to tool wear. Some studies reported favorable outcomes when various nanoparticle-enriched cutting fluids, such as reduced tool wear, improved surface quality, and lower cutting forces. However, limited research has explored the application of hybrid nanofluids^18–21^. The challenges associated with implementing trihybrid nanofluids are agglomeration, performance, and long-term stability at high temperatures^22–24^. Some studies have reported that adding two types of nanoparticles to a hybrid nanofluid can enhance their thermophysical, and tribological properties and machining characteristics^25–29^. Prior research is focused on hybrid nanofluid, very few research have investigated tri-hybrid in machining. This work aims to address this gap by investigating the performance of tri-hybrid nanofluids as cutting fluids in turning operations, cracking light on their potential benefits for the machining industry. This work focused on the performance of trihybrid nano cutting based on MWCNTAl_2_O_3_Gr for varying weight percentages in deionized water and corn oil in turning SS 304 steel.

## Materials and methods

**Fig. 1:**
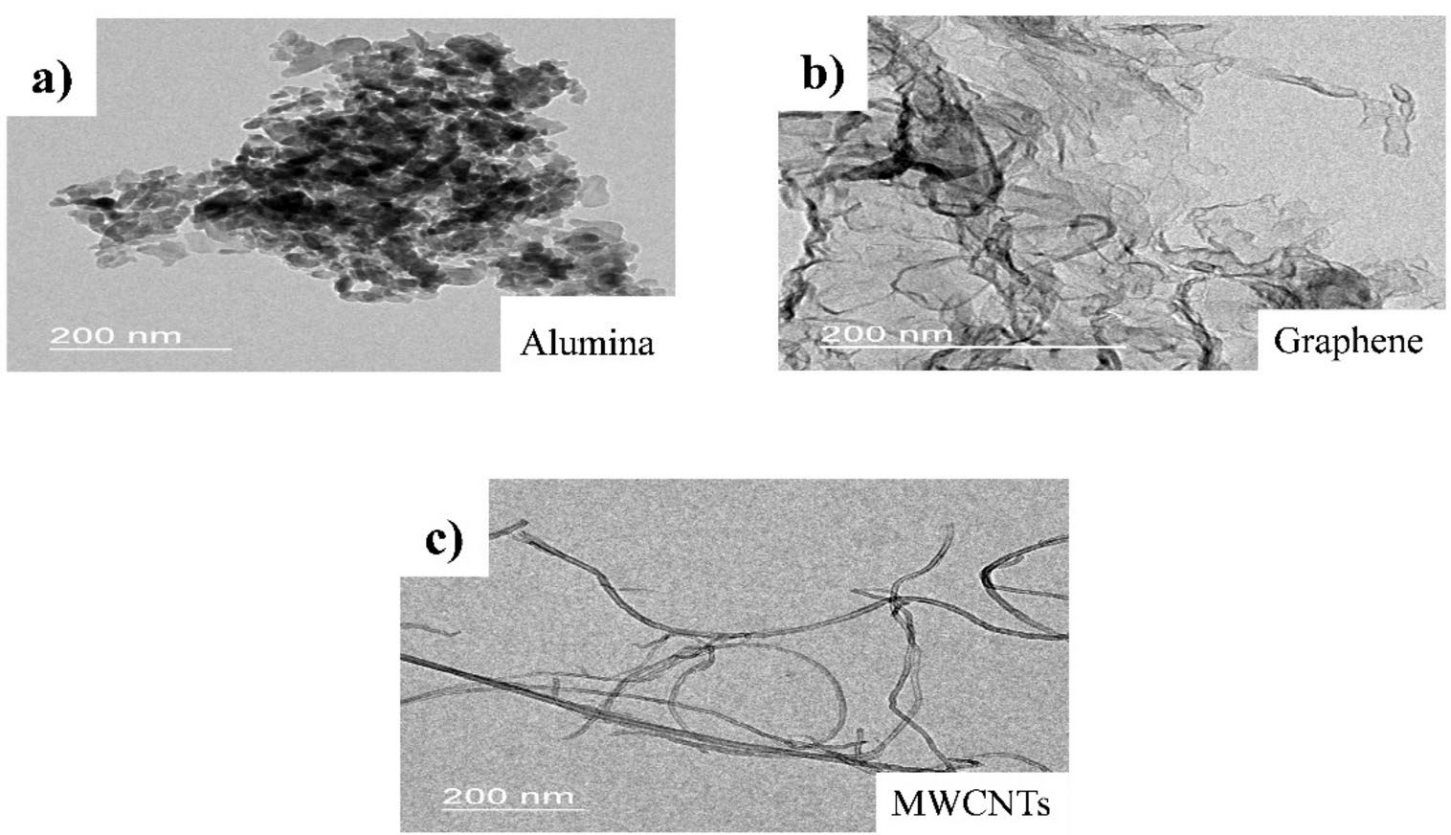
TEM images of additives, (**a**) Alumina; (**b**) Graphene; (**c**) MWCNTs.

**Fig. 2:**
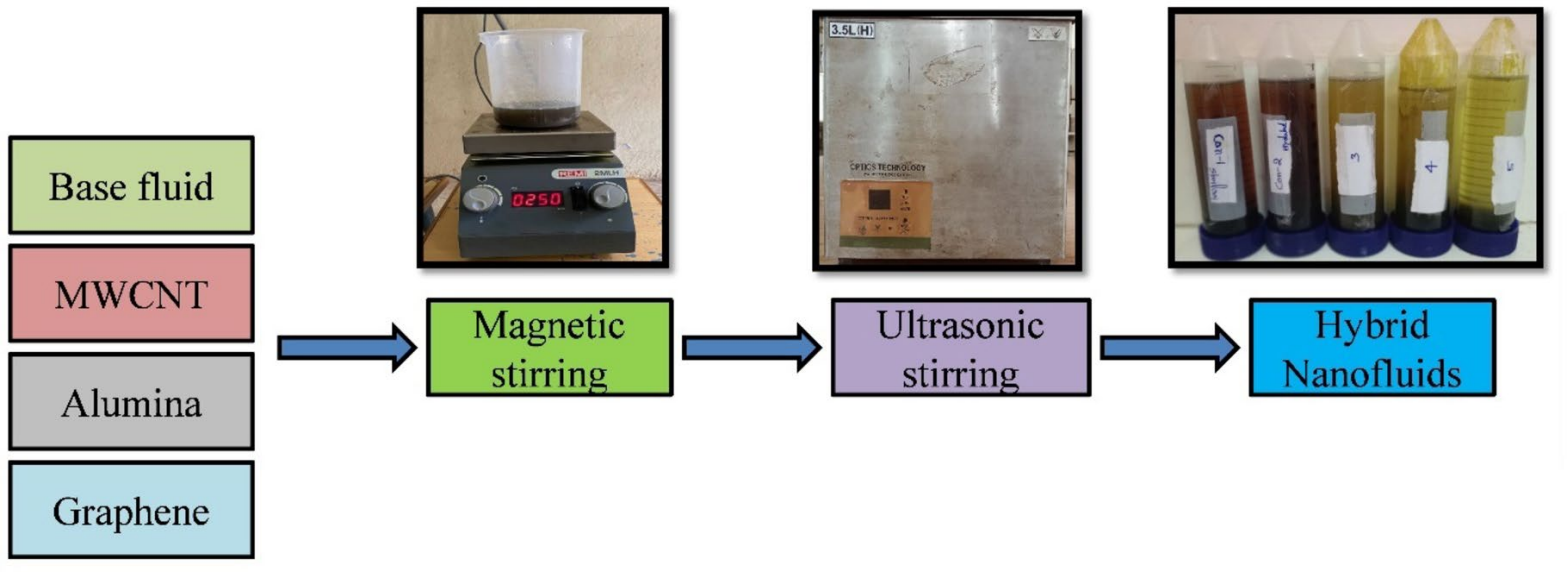
Preparation of tri hybrid nanofluids.

Newly developed MWCNTAl_2_O_3_Gr nanofluids with six weight concentrations ranging from 0.2 to 1.2 wt%. Equal composition of the MWCNTAl_2_O_3_Gr nanoparticles is considered. Corn oil and deionized water were mixed in 1:16 to disseminate these nanoparticles. Corn oil is selected due to several advantages such as biodegradability, high flash point and thermal stability. The nanoparticles were sonicated for 4 fours. In this study, a biodegradable oil-based cutting fluid is used as the base fluid, with nanoparticles of multi-walled carbon nanotubes (MWCNTs), alumina (Al₂O₃), and graphene (Gr) incorporated in equal proportions. These nanoparticles, with an average size of 35 nm, are selected for their superior thermal conductivity, tribological properties, and ability to enhance lubrication performance. Six different weight concentrations, ranging from 0.2 wt% to 1.2 wt%, are prepared to study the impact of nanoparticle concentration on machining efficiency. To achieve a well-dispersed and stable nanofluid, a three-step dispersion process is followed. Initially, vigorous manual stirring is performed for 30 min to break down large agglomerates and ensure preliminary mixing of the nanoparticles in the base fluid. This is followed by magnetic stirring for one hour further enhance nanoparticle distribution and promote interfacial interactions. Finally, ultrasonication is applied for 30 min to break down any remaining agglomerates and achieve a stable nanoscale suspension. Figure 1a–c shows TEM images of the additives and Fig. 2 represents the preparation methods of Tri hybrid nanofluids.

**Fig. 3:**
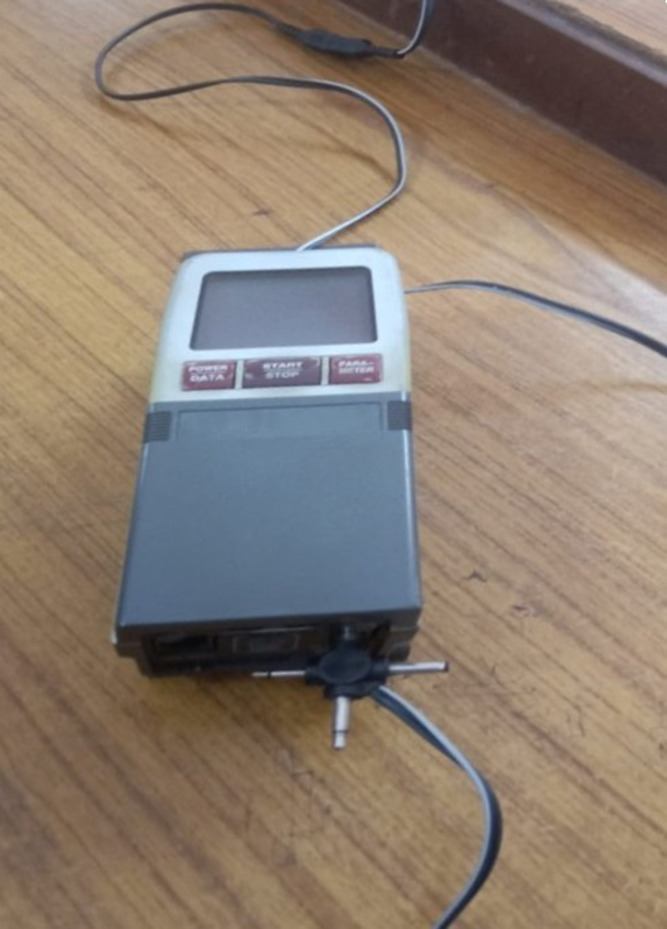
Surface roughness tester.

**Fig. 4:**
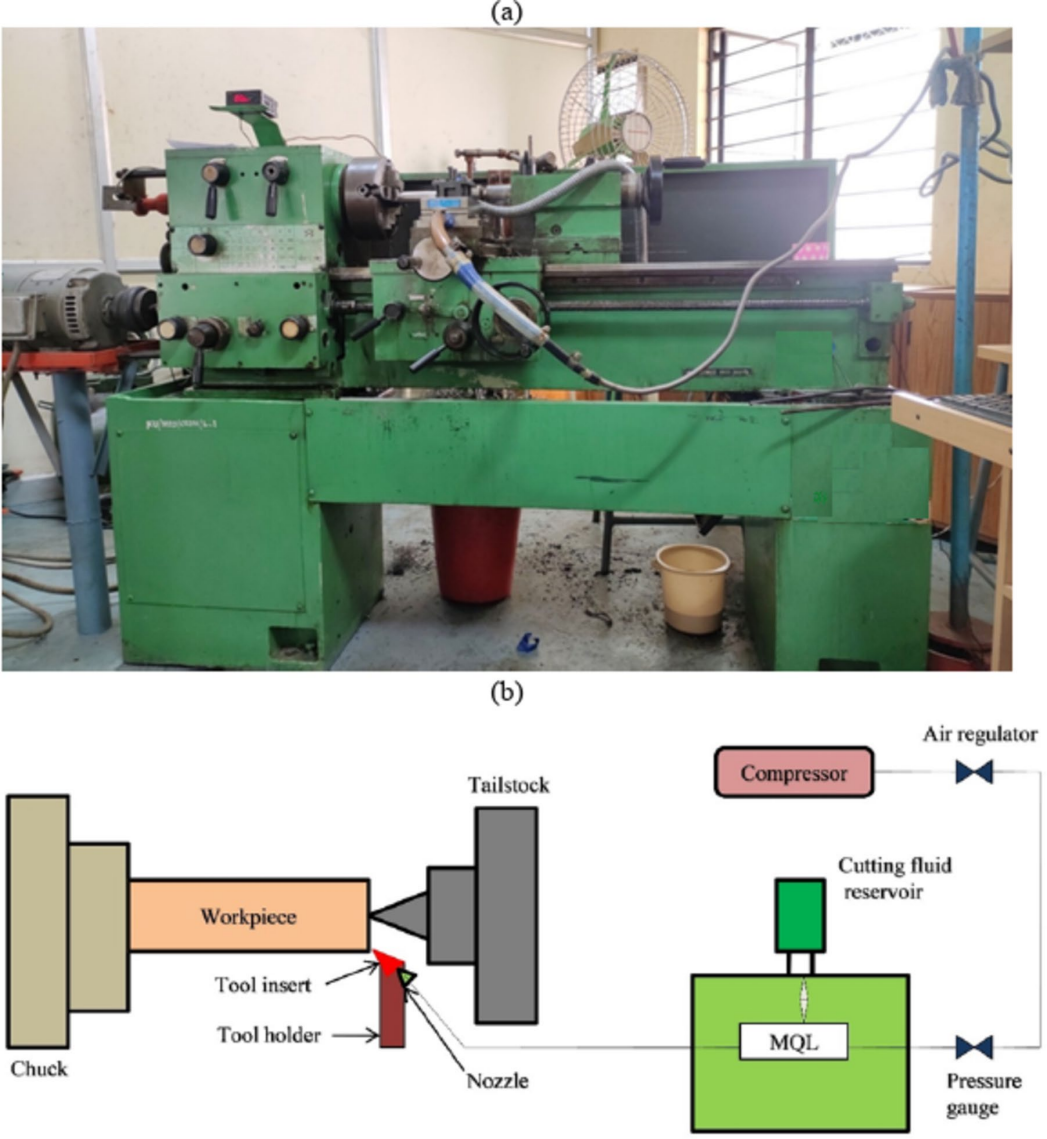
(**a**) Lathe Machine (Make: Kirloskar Turnmaster 35). (**b**). Schematic diagram of experimental setup.

An experimental matrix is developed considering five factors reported in Table 1. A central composite rotatable design method is employed with half factorial. The output parameters studied are surface roughness and cutting temperature. Turning tests are conducted on SS304 steel bars of 50 mm diameter. SS304 steel material is corrosion-resistant and has excellent strength and is widely used in the automotive and aerospace sectors. TiAlN-coated carbide tools are chosen for their exceptional thermal resistance, hardness, and wear protection, making them ideal for machining selected steel and MQL applications. A Turnmaster 35 Kirloskar Lathe Machine was used to run the experiments. Selection of cutting is made based on recommendations from the tool manufacturer, workpiece material and literature survey. MWCNTAl_2_O_3_Gr nanofluids were supplied with the help of the MQL system. The nozzle was positioned at 60° and 30 mm away from the workpiece. The flow rate and air pressure were set at 20 ml/min 5 PSI respectively. The surface roughness (Ra) of the machined part is measured using a surface roughness tester device (Make: Mitutoyo, model 178-561-12 A), shown in Fig. 3 and cutting temperature is recorded using an infrared thermometer (Make: Mitutoyo, model SJ 210P). The experimental setup is shown in Fig. 4a,b, and the machining parameters are demonstrated in Table 2.

**Table 1:** Factors and levels for the turning parameters of SS 304.

Factor	Parameter	Level				
		Low (− α)	High (− 1)	0	High (+ 1)	High (+α)
P	Cutting speed (RPM)	800	900	1000	1100	1200
Q	Feed (mm/min)	10	30	50	70	90
R	Depth of cut (mm)	0.3	0.45	0.6	0.75	0.9
S	MQL flow rate (ml/min)	20	30	40	55	60
T	Concentration (wt %)	0.25	0.50	0.75	1	1.25

**Table 2:** Machining parameters.

Workpiece	SS 304
Specimen size	50 mm diameter,200 mm long
Tool insert	TiAlN coated carbide
Coolant	MWCNTAl_2_O3Gr nanofluids in deionized water and corn oil
Coolant supply technique	Minimum quantity lubricant (MQL)
Machine	Turn master 35 centre lathe

## Results and discussion

### Contribution of independent variables to surface roughness and cutting temperature

The ANOVA is performed to study the relative significance of the process parameters and their interaction with a 95% confidence level. The F-values and P-value are used to obtain the significance criteria. Table 3 shows the ANOVA results for surface roughness and cutting. The p-values for the models is observed as less than 0.05, the terms involved have the significant impact on surface roughness and cutting temperature. The flow rate (Q) have significant impact on surface roughness and cutting temperature, followed by depth of cut. The death of cut is found most influencing parameter for cutting temperature followed by flow rate and conceteations. The F-value corresponding to feed (Q) is 472.790. The interaction effect of cutting speed (P) and feed (Q) on surface roughness was significant, with an F-value of 473.270 followed by the quadratic term R^2^.

**Table 3:** Independent variables contributions.

CCD	Probability ‘*P*’ value		Fishers’ ‘F’ value		I.V. contribution (%)	
	Dependent variables (D.V.) A: Surface roughness (µm) and B: cutting temperature (^o^C)					
Independent variables (I.V.) P: Speed Q: Feed R: Depth of cut S: Flow rate T: Concentration	A	B	A	B	A	B
Model	0.000	0.000	223.600	38.720	99.75%	98.60%
Linear	0.000	0.000	165.990	79.440	59.79%	34.37%
P	0.000	0.000	27.960	125.670	1.35%	0.28%
Q	0.000	0.027	472.790	6.490	0.22%	1.29%
R	0.000	0.049	33.440	4.870	8.09%	18.58%
S	0.000	0.000	75.230	241.260	49.90%	8.65%
T	0.000	0.021	230.650	7.280	0.22%	5.58%
Square	0.000	0.000	79.270	46.590	9.28%	23.84%
P*P	0.878	0.000	0.020	96.130	0.46%	0.19%
Q*Q	0.000	0.277	56.960	1.310	1.23%	0.13%
R*R	0.000	0.062	118.810	4.300	3.16%	2.84%
S*S	0.000	0.000	95.440	166.000	2.90%	19.24%
T*T	0.000	0.004	68.640	12.740	1.54%	1.45%
2-way interaction	0.000	0.000	137.570	31.720	30.69%	40.39%
P*Q	0.000	0.001	473.270	18.600	10.56%	2.37%
P*R	0.000	0.001	202.800	18.730	4.52%	2.38%
P*S	0.451	0.000	0.610	51.510	0.01%	6.56%
P*T	0.000	0.000	104.650	40.730	2.33%	5.19%
Q*R	0.343	0.000	0.980	29.100	0.02%	3.71%
Q*S	0.031	0.007	6.070	10.880	0.14%	1.39%
Q*T	0.000	0.353	141.740	0.940	3.16%	0.12%
R*S	0.000	0.000	37.160	40.580	0.83%	5.17%
R*T	0.000	0.001	105.690	23.350	2.36%	2.97%
S*T	0.000	0.000	302.750	82.810	6.75%	10.54%
Error					0.25%	1.40%
Total					100%	100%
R square (R^2^)	99.75%	98.60%				

### Interaction effects of independent variables on the surface roughness and cutting temperature

**Fig. 5:**
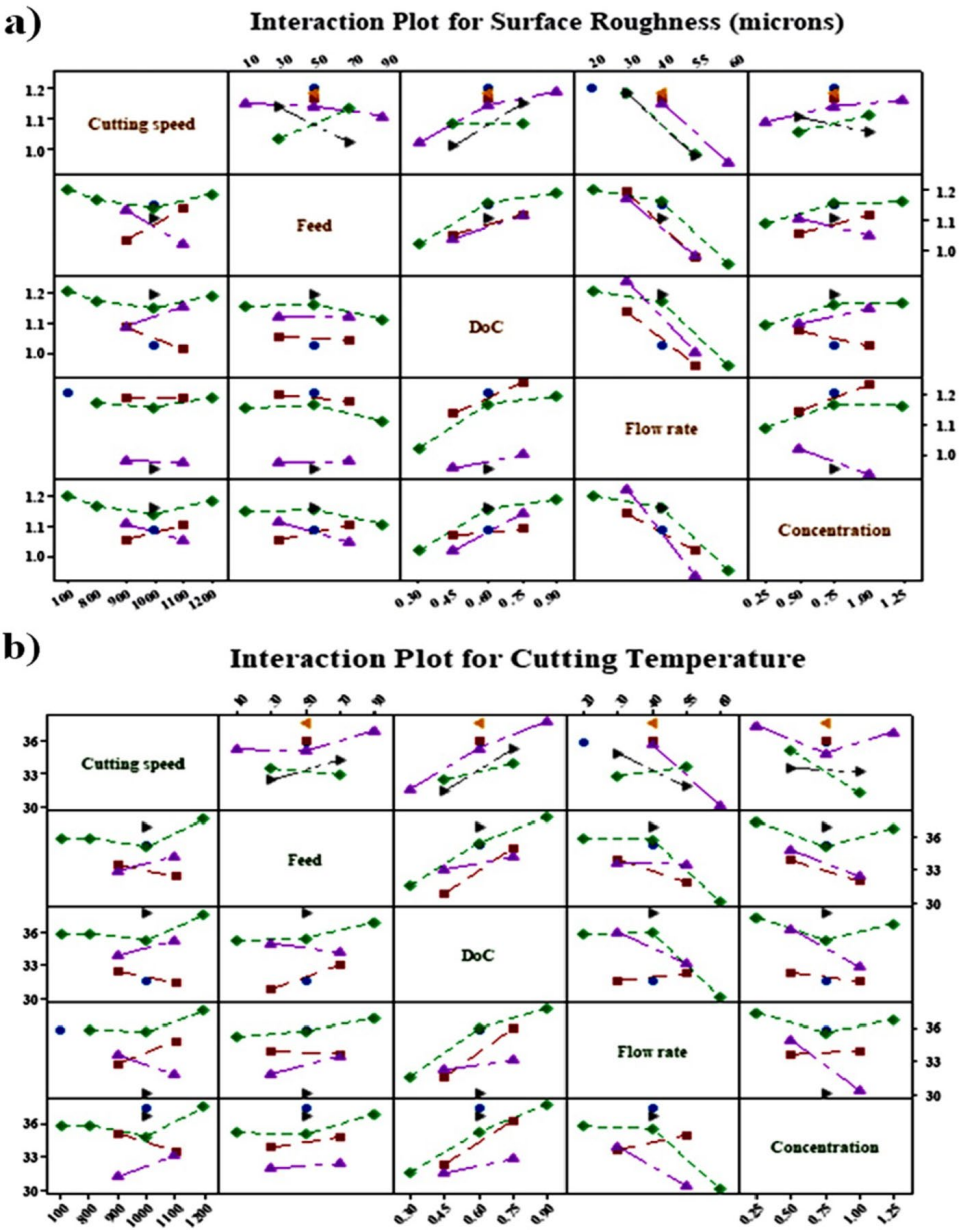
Independent variable interaction plot (**a**) surface roughness, and (**b**) cutting temperature.

The effects of the independent variables on the surface roughness and cutting temperature is shown in Fig. 5a,b. Figure 4a illustrates the interaction effect of input parameters on the output responses. The interaction plot helps to understand which factors and interactions are most important in determining the outcomes of interest. Figure 4a also shows that the speed and feed significantly affect the surface roughness. Furthermore, the flow rate and concentration have a significant combined effect. Similarly, the interactions between the speed and flow rate, flow rate and concentration, and depth of cut and flow rate seem to have the most significant effects on the cutting temperature (Fig. 4b).

### Regression analysis

The regression analysis is performed to develop predictive mathematical models for surface roughness and cutting temperature. The dependent parameters namely, cutting speed, feed, depth of cut, flow rate, and concentration are considered. The mathematical models are shown in Eqs. (1), (2).

1 $$\begin{aligned} Surface{\text{ }}Roughness{\text{ }}\left( {microns} \right) & = ~~ - 0.828{\text{ }} + ~0.000773~P{\text{ }} + ~0.03289~Q{\text{ }} - ~1.214~R \\ & + ~0.02620~S{\text{ }} + ~1.859~T{\text{ }} - ~0.000000~P*P{\text{ }} - ~0.000035~Q*Q{\text{ }} - ~0.9031~R*R \\ & - ~0.000226~S*S{\text{ }} - ~0.2471~T*T{\text{ }} - ~0.000027~P*Q{\text{ }} + ~0.002337~P*R{\text{ }} \\ & - ~0.000001~P*S{\text{ }} - ~0.001008~P*T{\text{ }} + ~0.000813~Q*R{\text{ }} + ~0.000024~Q*S \\ & - ~0.005862~Q*T{\text{ }} - ~0.00795~R*S{\text{ }} + ~0.6750~R*T{\text{ }} - ~0.013618~S*T \\ \end{aligned}$$

2 $$\begin{aligned} Cutting{\text{ }}Temperature~\left( {^{o} C} \right)~~ & = ~~28.36{\text{ }} - {\text{ }}0.09270{\text{ }}P{\text{ }} - {\text{ }}0.2180{\text{ }}Q{\text{ }} + {\text{ }}26.2{\text{ }}R{\text{ }} + {\text{ }}2.654{\text{ }}S{\text{ }} - {\text{ }}18.69{\text{ }}T \\ & + {\text{ }}0.000030{\text{ }}P*P{\text{ }} + {\text{ }}0.000301{\text{ }}Q*Q{\text{ }} - {\text{ }}9.72{\text{ }}R*R{\text{ }} - {\text{ }}0.01685{\text{ }}S*S \\ & + {\text{ }}6.02{\text{ }}T*T{\text{ }} + {\text{ }}0.000300{\text{ }}P*Q{\text{ }} + {\text{ }}0.04019{\text{ }}P*R{\text{ }} - {\text{ }}0.000770{\text{ }}P*S \\ & + {\text{ }}0.03556{\text{ }}P*T{\text{ }} - {\text{ }}0.2505{\text{ }}Q*R{\text{ }} + {\text{ }}0.001826{\text{ }}Q*S{\text{ }} - {\text{ }}0.0270{\text{ }}Q*T \\ & - {\text{ }}0.4701{\text{ }}R*S{\text{ }} - {\text{ }}17.95{\text{ }}R*T{\text{ }} - {\text{ }}0.4030{\text{ }}S*T \\ \end{aligned}$$

**Fig. 6:**
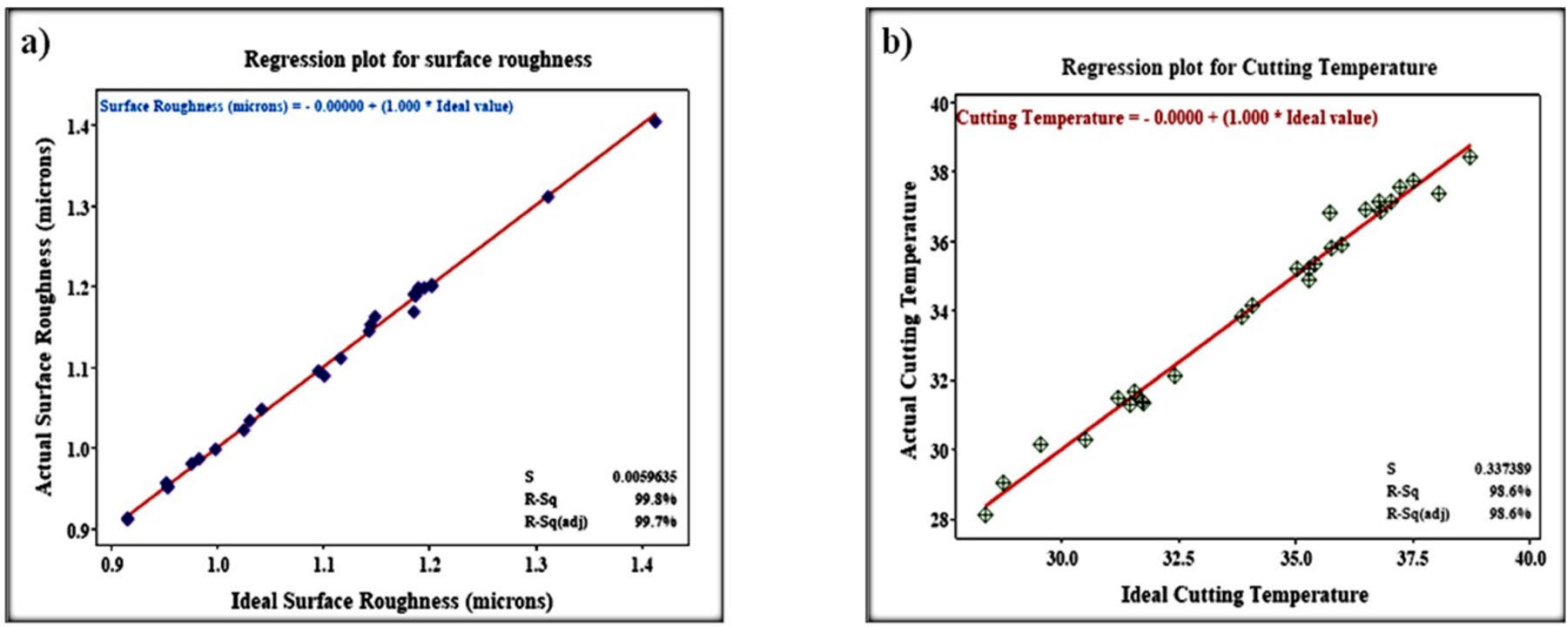
Regression plots (**a**) surface roughness, and (**b**) cutting temperature.

These models are verified through the confirmation test using normal probability plots. The regression coefficient (R^2^) value is used to evaluate the quality of models. The R^2^ value near to one is preferred which implies residuals are minimum and the model better fits data. The R^2^ value of 0.87 and 0.92 is observed for the surface roughness model and cutting temperature. It can be concluded that the factors considered and their interaction have significance. Using a residuals plot (Fig. 6a,b) the reliability of the models was verified. The model makes an excellent prediction since the residuals lie near a straight line, indicating the error is normally distributed.

### Composite desirability condition

**Fig. 7:**
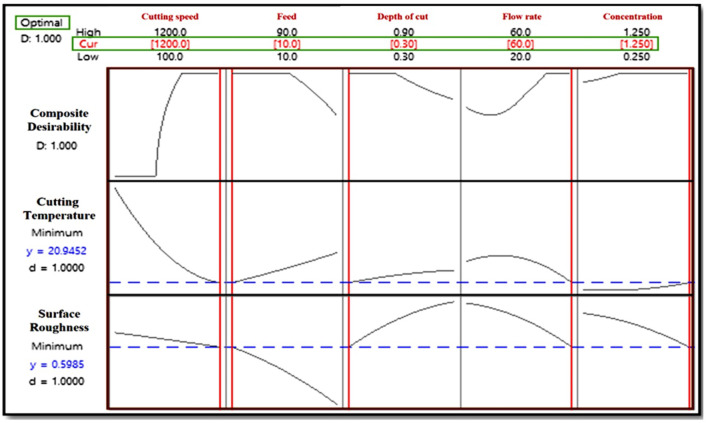
Composite desirability plot.

A composite desirability plot (Fig. 7) provides a systematic framework for integrating multiple criteria into a single measure, enabling decision-makers to make more informed and balanced decisions across complex decision-making scenarios. The overall index that is determined by combining each answer variable and processing it through a geometric mean is called the composite desirability (D), which determines the optimal conditions for simultaneously optimizing each response variable. The desirability (D) values for the cutting temperature and surface roughness are 1, which indicates that the procedure was highly optimized because these indices are extremely close to the ideal state.

### Confirmation test

Table 4 shows the comparative results obtained for the selected cutting conditions. The obtained optimal cutting parameters are found as speed 1200 rpm, feed 10 mm/min, depth of cut: 0.3 mm, flow rate: 60 g/s, and concentration: 1.25 wt%.

**Table 4:** Confirmation study based on CCD’s composite desirability.

Optimum Independent variables Speed: 1200 rpm; Feed: 10 mm/min; Depth of cut: 0.3 mm; Flow rate: 60 g/s; and Concentration: 1.25 wt%					
Surface roughness (µm)			Cutting temperature (^o^C)		
Predicted	Experimental	Residue	Predicted	Experimental	Residue
0.5985	0.652	0.0535	20.9452	21.5	0.548

## Artificial neural network

**Fig. 8:**
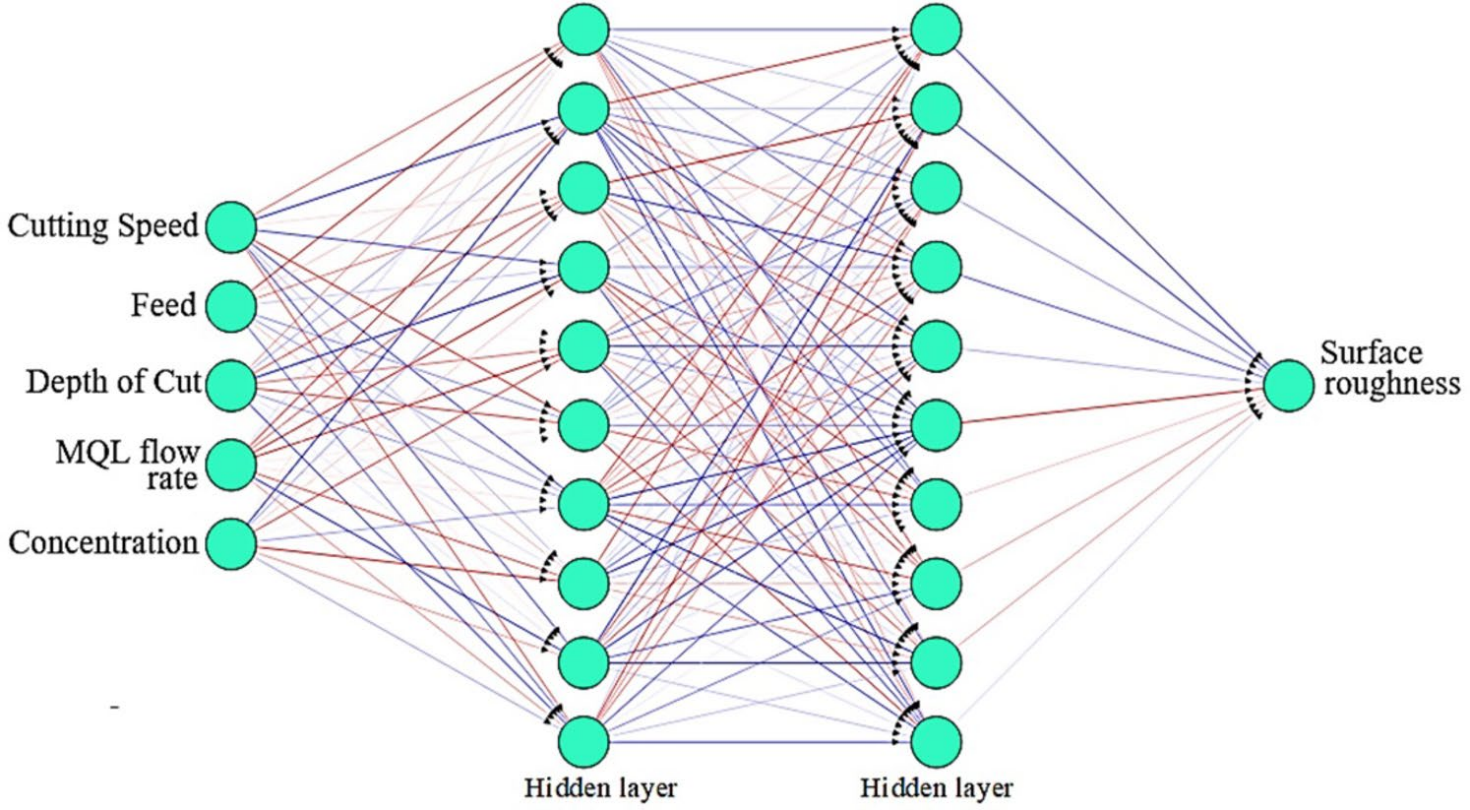
ANN architecture for surface roughness.

**Fig. 9:**
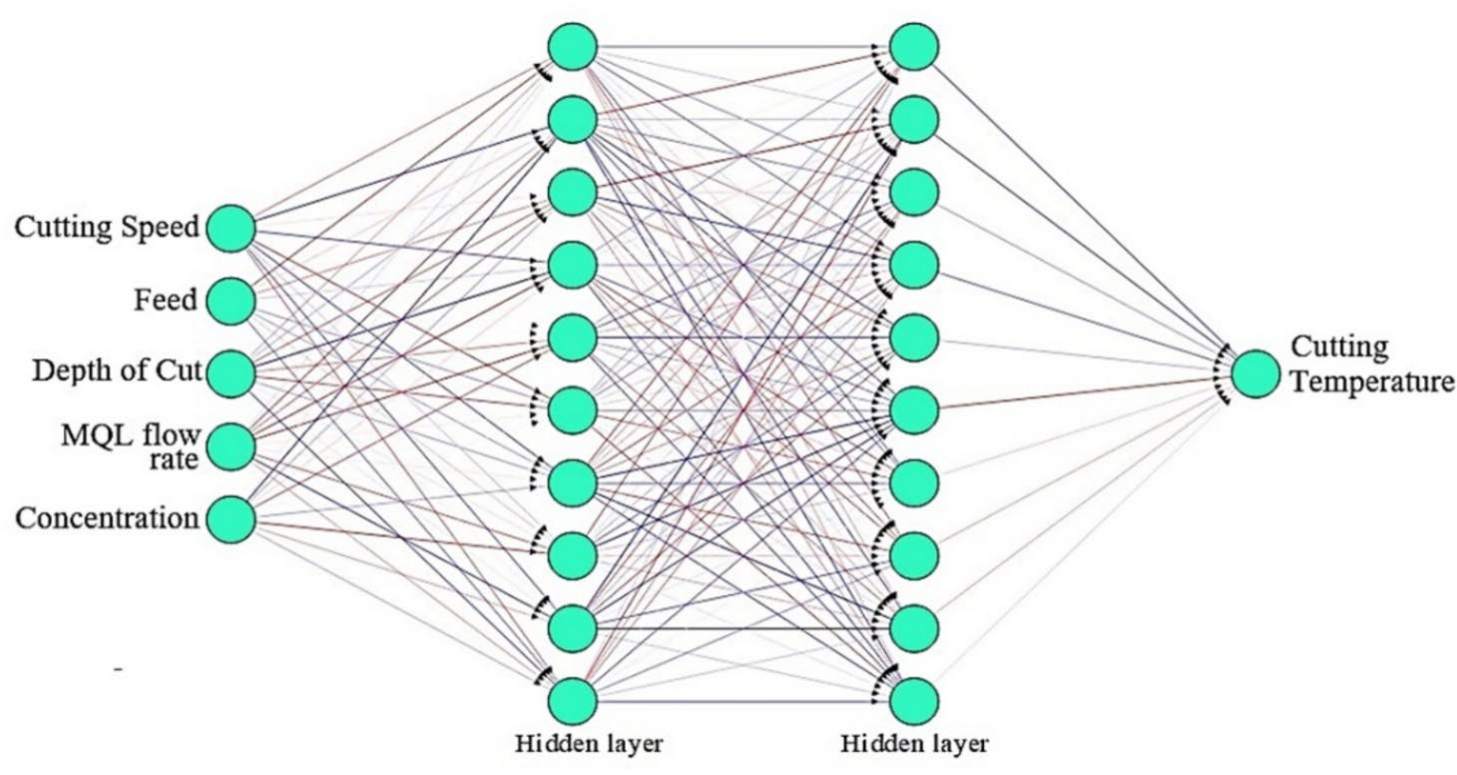
ANN architecture for the cutting temperature.

Artificial neural networks (ANNs) play a crucial role in optimizing machining processes, supported by the MQL method^30^. The MQL method involves the use of a minimal amount of lubricant during machining to reduce the environmental impact, risks, costs, and health hazards associated with traditional flood cooling methods^31,32^. ANNs can be trained to model the complex relationships between machining parameters (such as the cutting speed, feed rate, and depth of cut) and performance metrics (surface roughness and cutting temperature) under MQL conditions. ANN-based models reduce the need for extensive experimental trials by simulating and predicting the effects of different MQL parameters^33^. In this study, ANNs are integrated into control systems to provide adaptive control for MQL machining processes. This allows for real-time adjustments of machining parameters based on changing conditions, ensuring consistent performance and quality^34^. The ANN model was developed in MATLAB version R2021 using a feedforward back-propagation machine learning algorithm^35^. The ANN architecture has two hidden layers with 150 neurons on each layer. Typically, this approach uses less computing time but requires more memory. The mean squared error (MSE) is used to assess the effectiveness of the ANN^36^. The optimal selection of the number of neurons and hidden layers is imperative during the training of an ANN to achieve improved prediction accuracy. An illustration of the ANN architecture for surface roughness is shown in Fig. 8. A similar ANN architecture is also developed for the cutting temperature, as shown in Fig. 9. The training of an ANN model to forecast the surface roughness and cutting temperature remains the main focus of this study.

**Fig. 10:**
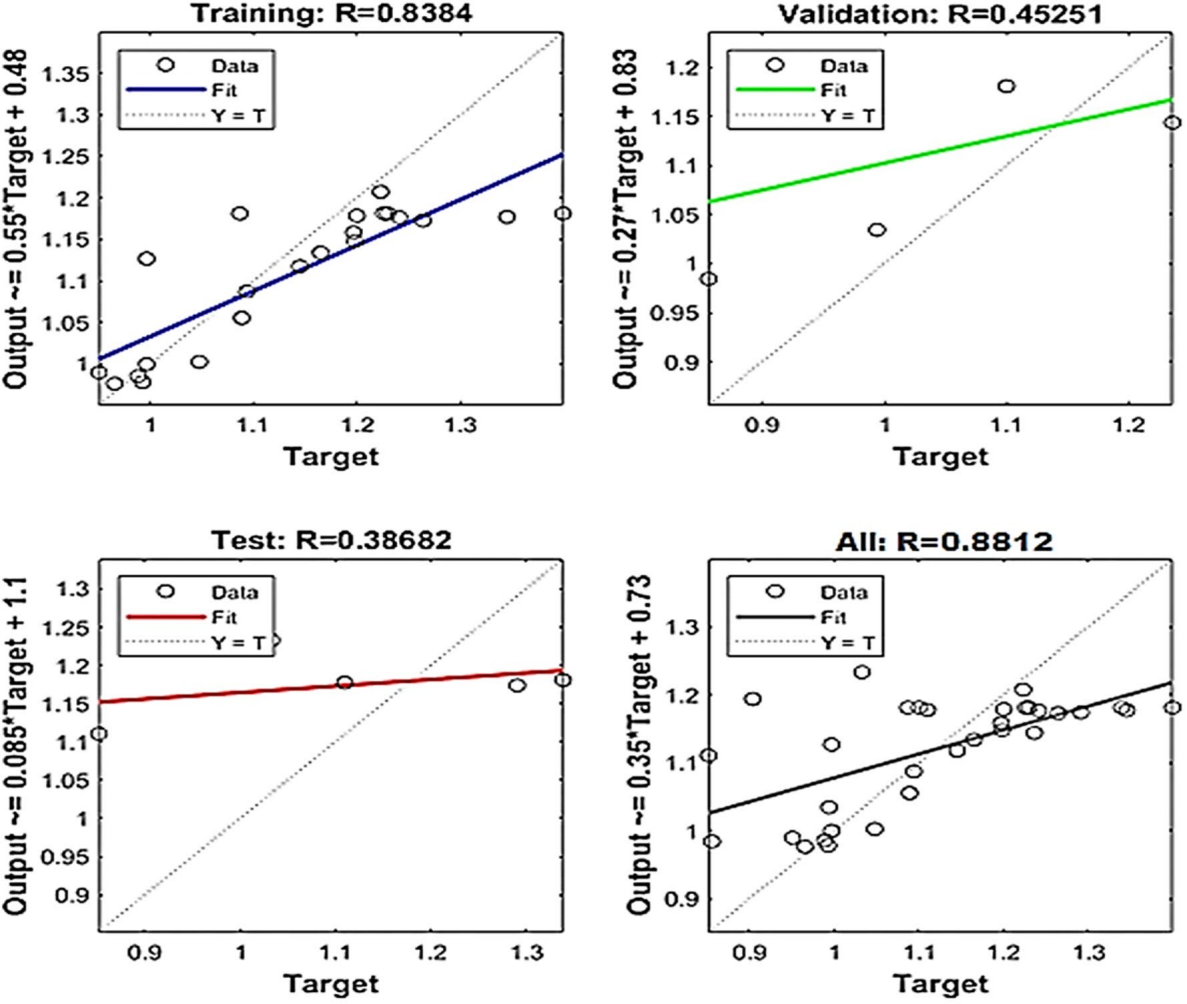
Regression graph for surface roughness.

**Fig. 11:**
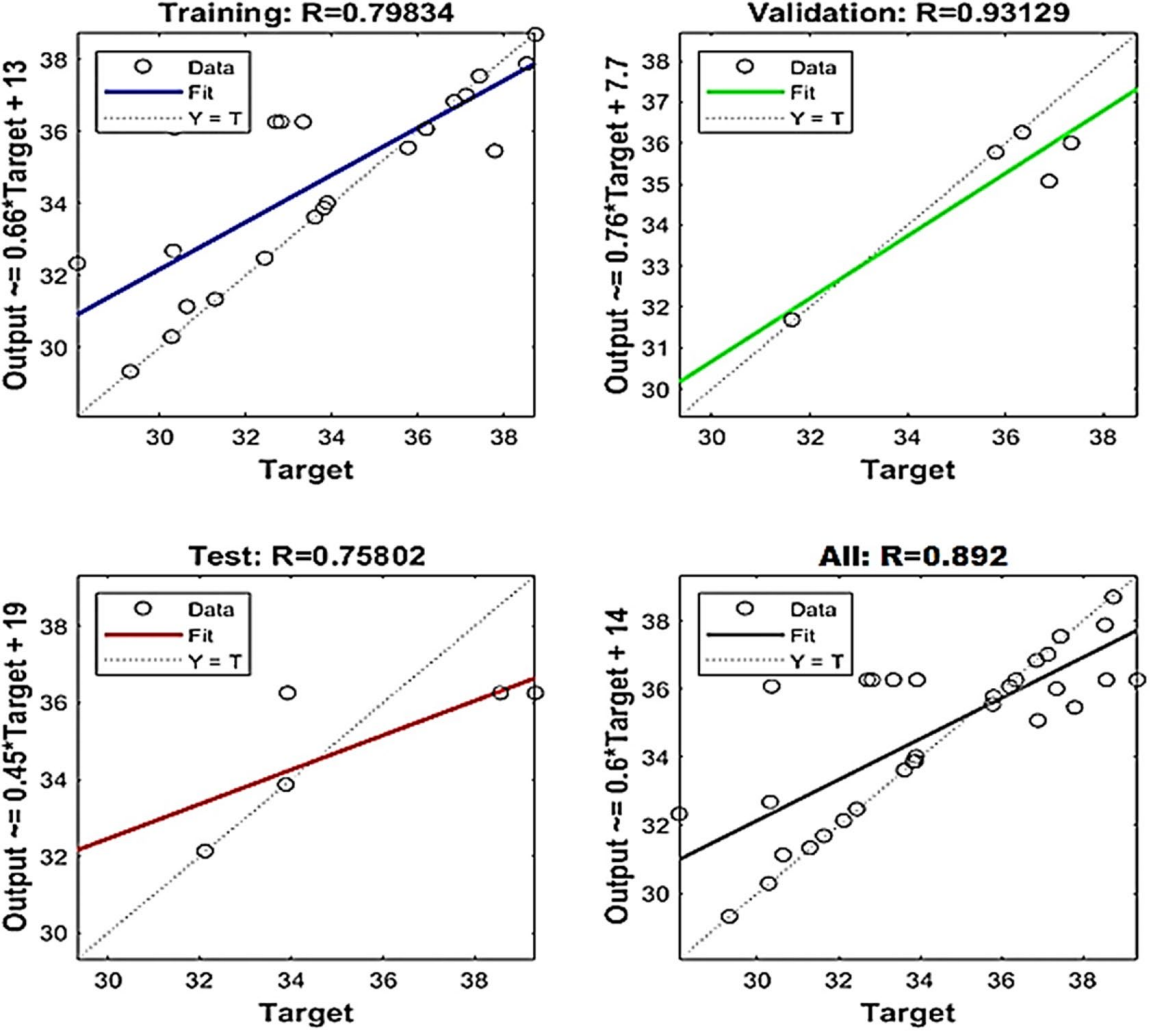
Regression graph for cutting temperature.

The network performance is evaluated using correlation coefficient value (R). The regression plots are reported in Figs. 10 and 11, respectively. Surface roughness and cutting temperature have correlation values 0.88 and 0.89 respectively. These values are near to 1 which implies good agreement between ANN predictions and the experimental results.

## Conclusion

This study employs MQL method and tri-hybrid nanofluids to examine machining performance at high speed using coated carbide cutting tool. The equal composition of the MWCNTAl_2_O_3_Gr nanoparticles are considered. The experimental results indicate a 76% reduction in temperature and a 16% improvement in surface roughness, demonstrating enhanced surface quality. Additionally, predictive models were developed using Artificial Neural Networks (ANN) and regression analysis. ANOVA revealed that the surface quality is considerably impacted by the cutting speed (P) and feed (Q) followed by the quadratic term R^2^. The developed correlations exhibit strong agreement with experimental data, with regression analysis and ANN yielding deviations of 1.13% and 3.14%, respectively. The study concludes that ANN predictions are more accurate than regression-based forecasts.

## Data Availability

All data generated or analyzed during this study are included in this published article.
